# Genomic diversification between aposematic and cryptic phenotypes of *Oophaga granulifera* (Anura: Dendrobatidae)

**DOI:** 10.1038/s41598-026-66654-3

**Published:** 2026-08-25

**Authors:** Anaisa Cajigas Gandia, Heike Pröhl, Julia Metzger, Ariel Rodríguez

**Affiliations:** 1 Institute of Zoology, University of Veterinary Medicine of Hannover, Bünteweg 17, 30559 Hannover, Germany; 2 Institute of Animal Genomics, University of Veterinary Medicine of Hannover, Bünteweg 17p, 30559 Hannover, Germany

**Keywords:** Coloration, Genes, *Oophaga*, Polymorphism, Natural selection, Transcriptomics, Ecology, Ecology, Evolution, Genetics, Molecular biology, Zoology

## Abstract

Aposematism and crypsis are contrasting antipredator strategies widespread across animal taxa, yet their genetic basis remains poorly understood in amphibians. Aposematic species advertise toxicity through conspicuous coloration, whereas cryptic species rely on concealment to avoid predators. The granular poison frog, *Oophaga granulifera*, with allopatric aposematic (red) and cryptic (green) morphs, provides an excellent system to explore the molecular mechanisms underlying transitions between these strategies. We used a comparative genomic approach, mapping RNA-seq reads to the *Oophaga sylvatica* reference genome and a de novo assembled *O. granulifera* superTranscriptome, to characterize its population genetics and detect genes with single nucleotide polymorphisms under positive selection. We identified two main genetic clusters with strong divergence between green and red populations. We found 283 SNPs showing signatures compatible with positive selection using the reference genome and 749 SNPs using the reference superTranscriptome, indicating greater sensitivity of the latter approach in capturing species-specific genetic variation. Some of these SNPs occur in coloration-related genes, including genes involved in melanin synthesis (*DVL2*, *FZD7*, *GNAI1*), pteridine synthesis (*MYCBP2*), iridophore development (*IMPDH1*, *PSAT1*), carotenoid metabolism (*BCO1*, *RARG*, *RETSAT*), and pigmentation variation. Our findings highlight the complex genetic basis of color diversity in anurans and the value of generating species-specific genomic resources for non-model organism’s research.

**Supplementary Information:**

The online version contains supplementary material available at https://doi.org/10.1038/s41598-026-66654-3.

## Introduction

Animal coloration is a long-standing focus in evolutionary biology due to its crucial role in physiological functions, such as thermoregulation and UV protection, and ecological interactions like sexual selection and predator avoidance^1^. Aposematism and crypsis are two anti-predator strategies linked to coloration. Aposematic species display bright warning colors that signal toxicity or unpalatability, to deter predators^2^. In contrast, cryptic species lack such defenses and depend on camouflage or disruptive patterns to blend into their environment, to avoid detection^3^. Predators can learn to associate vivid colors with negative experiences, granting aposematic species a survival advantage over cryptic ones, which rely entirely on remaining unnoticed. Both strategies are widespread and well-documented across invertebrates and all major vertebrate groups^1,2,4,5^.

Coloration as a warning signal varies widely, not only among species but also across populations of the same species^6^. This variation is especially striking in dendrobatid poison frogs, native to the Neotropics, where both aposematic and cryptic strategies are present among populations of a single species^7^. For instance, the strawberry poison frog *Oophaga pumilio* displays over 15 color morphs across populations in Bocas del Toro archipelago, Panama^8^.

The mechanisms underlying color-based anti-predator strategies in amphibians remain poorly understood. In frogs, coloration arises from a multicellular origin, with different skin cell types producing and storing multiple pigments (dermal chromatophore unit^9^). However, the amount and distribution of pigments is regulated by gene networks involving genes not only related to coloration but to different metabolic pathways^10^. Several evolutionary and ecological processes have been proposed as drivers of color diversity in dendrobatid frogs. These include sexual selection and predation pressure in *O. pumilio*^11,12^, hybridization in *Oophaga histrionica*^13^, and genetic drift acting in small populations of *Adelphobates galactonotus*^14^ and *O. pumilio*^15^.

Color polymorphic species like dendrobatid frogs offer an exceptional opportunity to investigate the genomic basis of phenotypic variation. By employing high-throughput sequencing techniques, it is possible to identify genes and pathways linked to color polymorphism, providing insights on its underlying evolutionary mechanisms^10,16^. Despite the promise of these methods, working with non-model organisms, which often lack reference genomes, introduces significant challenges^17^. In *Oophaga* frogs, the existing genomic resources are insufficient for full genome scans^18^. To overcome this, and limit costs, the use of reduced representation sequencing (RRS) methods that screen a fraction of the genome, such as transcriptome sequencing (aka RNA-seq) offer an alternative^19^. RNA-seq captures genome and transcriptome diversity from expressed genes and enables the discovery of molecular markers such as Single-Nucleotide Polymorphisms (SNPs), useful for studying color variation^20^.

The granular poison frog *Oophaga granulifera* is a brightly colored species distributed along the Pacific versant of Costa Rica. Previous phylogeographic studies have revealed deep intraspecific divergence accompanied by recent color diversification, supporting the existence of distinct northern and southern lineages rather than separate species^21^. Northern populations are predominantly dull green, whereas southern populations are brightly red, with intermediate phenotypes in the transition area. Experiments with clay models in this species indicate that predators exert a divergent selection on these phenotypes, with bird attack rates following the inverse of the coloration distribution (i.e. avoidance of local phenotypes), and lizard predation selecting against the red phenotype in the North^22^. Contrary to classical predictions, toxicity assays, spectral reflectance measurements, and phylogenetic reconstruction have revealed that the less conspicuous green color morphs evolved from a more conspicuous red ancestor and are more toxic than the red phenotypes^23^. In this study, we report on population genetics and evidence of selection at the gene level in two divergent color phenotypes of *O. granulifera.* Assuming that predator pressure and sexual selection are important selective forces driving the observed phenotypic divergence^22,24^, we aimed to identify genes showing signatures of divergent selection between the two color phenotypes. To overcome known limitations of transcriptome scans we implemented two alternative approaches: mapping RNA-seq reads against the reference genome of the closely related *Oophaga sylvatica* (little devil poison dart frog) and mapping the reads to a de novo assembled superTranscriptome (i.e. a non-redundant transcriptome assembly) of *O. granulifera*. This strategy allows a more comprehensive identification of genes, and the comparison of results will reveal the pros and cons of each method to uncover genotype-phenotype associations in non-model organisms with limited genomic resources.

## Materials and methods

### Data collection

**Fig. 1:**
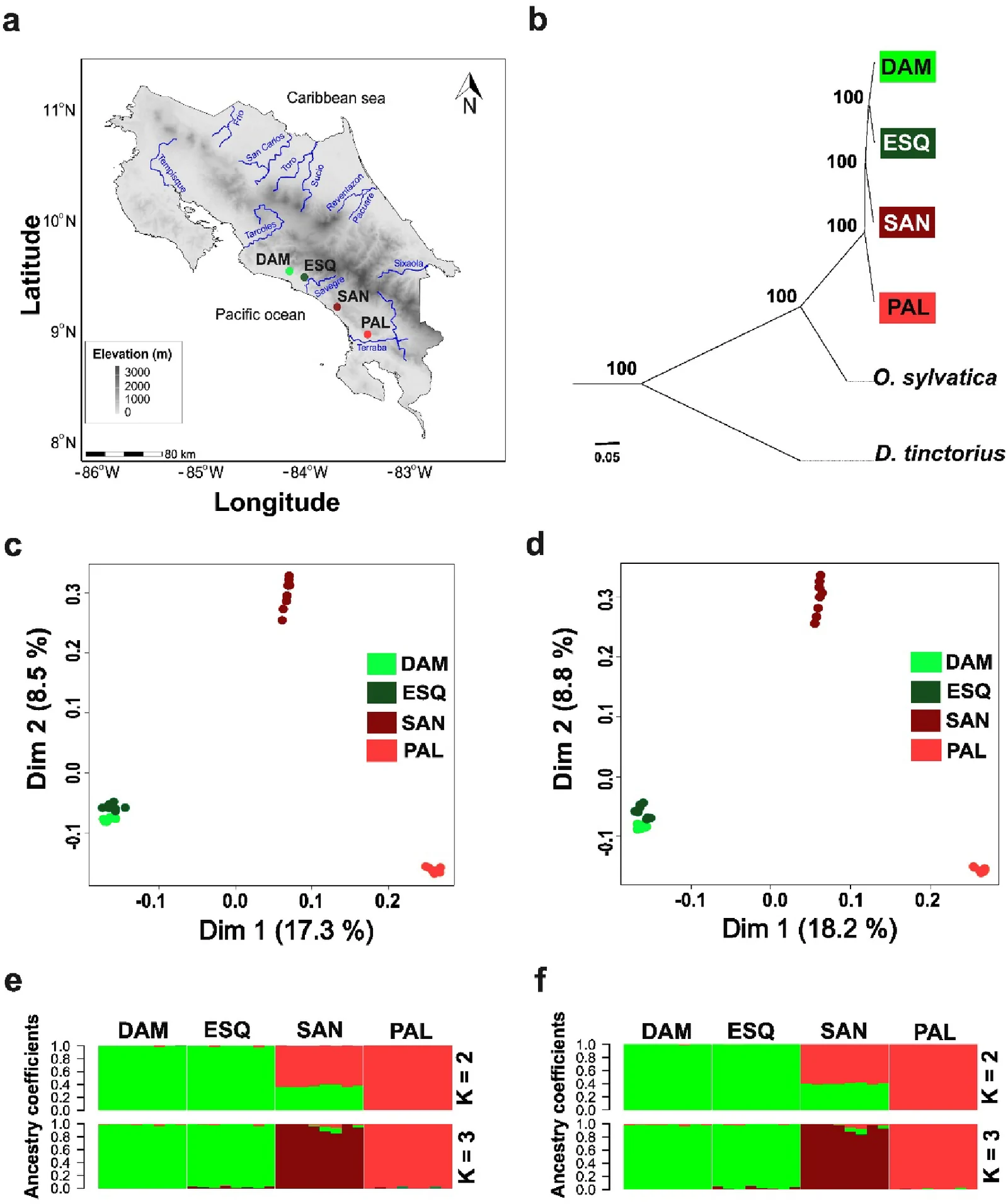
Population genetic structure of *O. granulifera* using the full datasets. (**A**) Sampled localities of *O. granulifera* in Costa Rica: dot colors represent the color morph of the frogs in the respective populations: green for San Antonio de Damas (DAM) and Esquipulas de Quepos (ESQ), and red for San Josecito (SAN) and Palmar Norte (PAL). (**B**) Coalescent phylogenetic tree showing the relationships among populations, and two outgroups: *O. sylvatica* and *D. tinctorius.* (**C**) Principal Component Analysis: genome. (**D**) Principal Component Analysis: superTranscriptome. (**E**) Admixture analysis: genome. (**F**) Admixture analysis: superTranscriptome. Points in (**C**) and (**D**) represent individuals, eight per population. Genome (*N* = 142 234 SNPs), superTranscriptome (*N* = 208 993 SNPs). In A) Costa Rica landmass limits, elevation and river data were obtained from https://diva-gis.org.

We used available RNA-seq data from skin, liver and eyes of *O. granulifera* (NCBI SRA Bioproject PRJNA1234489, accession numbers: SRR32643849 - SRR32643922). These samples cover the two extremes of the color variation in *O. granulifera* across four localities (Fig. 1A), two from the south representing the red morph: Palmar Norte (PAL: 8.976° N, 83.440° W; elevation: 365 m; *N* = 8 males) and San Josecito (SAN: 9.206° N, 83.758° W; elevation: 241 m; *N* = 8 males), and two from the north representing the green morph: San Antonio de Damas (DAM: 9.546° N, 84.190° W; elevation: 144 m; *N* = 8 males) and Esquipulas de Quepos (ESQ: 9.493° N, 84.046° W; elevation: 225 m; *N* = 8 males). Throughout this study, we refer to the green populations as cryptic and the red populations as aposematic based on previous work on *O. granulifera*^25,26^. However, we acknowledge that conspicuousness and warning signaling are context dependent and may vary with predator perception, toxicity, and local environmental conditions^12,26,27^. As required for some analyses, we added two samples of *Dendrobates tinctorius* (NCBI SRA Bioproject PRJNA801674, accession numbers: SAMN25373888 - SAMN25373889), a species belonging to the sister genus of *Oophaga*^28^, as an outgroup. Additional details on sample characteristics, the phenotypes and associated metadata can be found in Supplementary Table S1, and in^26,29,30^.

### SuperTranscriptome assembly and annotation

We used an available reference transcriptome of *O. granulifera* (DDBJ/EMBL/GenBank accession number: GLKT00000000) to generate a species-specific superTranscriptome suitable for genotype calling. SuperTranscriptomes constitute a valid alternative for representing the transcriptome sequences of a species without redundancy as each super-transcript contains the concatenated isoforms of each expressed gene^31^. We used Lace software *v*. 3.17^31^ to concatenate the ORF-containing transcripts in the reference transcriptome into a set of super-transcripts. The gene-to-transcript table, obtained during the original transcriptome assembly (see details in^30^) was used as input to Lace to guide the concatenation of transcripts and later used as annotation. The superTranscriptome of *O. granulifera* provides a valuable new resource for amphibian genomics. This addresses a key limitation: the high genetic distance between *O. sylvatica* and *O. granulifera*^32^ may result in the loss of important genetic information when mapping *O. granulifera* reads against the genome of *O. sylvatica.*

### RNA reads mapping, variant calling and filtering

The genome assembly of *O. sylvatica* (NCBI accession number: GCA_033576555.1), and its annotation^33,34^ were used as reference to align all RNA-seq reads using the two-pass mode of STAR and default settings^35^. A similar procedure was used for the superTranscriptome approach but setting *--outFilterScoreMinOverLread* and *--outFilterMatchNminOverLread* to 0.3 to filter out low-quality alignments (i.e. reads with less than 30% of their length correctly mapped). These additional filters in the superTranscriptome approach help to compensate for the structural differences between genome-based (i.e. canonical exon-intron structures) and superTranscriptome-based (i.e. only exonic regions) alignments, ensuring alignment quality is maintained in both contexts.

Variants were called using GATK software^36^ following the best practices workflow and recommendations for RNA-seq based haplotype calling^37^. The pipeline included: identifying duplicate reads, merging tissue reads per individual, splitting exon spanning reads and clip sequences overhanging the intronic regions, variant calling per individual, joint variant calling of all individuals, and quality filtering to include only bi-allelic SNPs with Fisher strand values (FS) > 30.0 and quality by depth values (QD) < 2.0. The resulting genotype file was further filtered using VCFtools *v.* 0.1.16^38^ to retain genotypes with a minimum depth (minDP ≥ 3), sites with minor allele frequency (maf ≥ 0.05), and sites with a maximum of 25% of missing data (max-missing ≥ 0.75). The remaining SNPs were then pruned for linkage disequilibrium (LD) in PLINK *v.* 2.0^39^ using the “--indep-pairwise” option, applying the same parameters for both the genome and the superTranscriptome (window size: 50, variant count: 1, squared correlation threshold: 0.7). In the genome-based SNPs calling, from an initial set of 13 399 434 variants, only 142 234 SNPs remained after filtering, whereas in the superTranscriptome-based SNPs calling, from originally 1 820 486 variants, 208 993 SNPs remained. These two sets of SNPs were used to conduct population genetics analyses and selection scans.

### Population genetics analysis

Population structure and genetic diversity analyses were performed using the *SambaR* package^40^ in R *v.* 4.4.1^41^. According to the package manual, different filter configurations should be applied in correspondence with the type of analysis. However, because our dataset was previously filtered, we applied the filter configuration suggested by the authors to retain all our data^42^. To do so, we opted for high (1) *snpmiss* (i.e. maximum allowed proportion of missing data per SNP) and *indmiss* (i.e. maximum allowed proportion of missing data per sample) values. The min-mac (i.e. minimum allowed number of minor allele copies per SNP) was set to 0 since from previous filtering we only kept bi-allelic SNPs, and the min-spacing filter (i.e. minimum distance between adjacent SNPs when thinning the data) was also set to 0, since LD filtering was already performed with PLINK. Population structure analyses included Principal Component Analysis (PCA), and LEA Admixture analysis (nruns = 50, kmin = 1, kmax = 4: in correspondence with the number of studied populations). The most likely *K* value was estimated using the cross-entropy criterion. Using SNPs under selection can bias population structure analyses, as they reflect adaptive rather than neutral processes^43,44^. Whole-genome sequencing could mitigate this issue by offering broader data^45,46^, but it was not feasible in our case. Neutral markers are often preferred for their unbiased representation^47^, and methods exist to exclude selected SNPs^48^, although this approach is debated because adaptive SNPs may also inform population structure^49^. Recent studies suggest comparing results from both neutral and selected SNPs^50^ and using statistical corrections like Bayesian methods or PCA^51,52^. To account for the potential effects of selection on population structure, we re-ran the structure analysis using as input a filtered vcf file containing only the neutral loci identified by BayeScan (see below), comparing PCA and Admixture outcomes using full versus filtered datasets from the genome and superTranscriptome. In this case, sample size for the genome and superTranscriptome was 51 195 SNPs and 70 187 SNPs respectively. We assessed genetic diversity by analyzing nucleotide diversity (π), and the Watterson estimator theta (Θ)^53^. To quantify population genetic divergence, we used Nei´s distance^54^, and Weir & Cockerham 1984 *Fst*^55^. These analyses were performed with the full dataset for the genome (*N* = 142 234 SNPs) and superTranscriptome (*N* = 208 993 SNPs) approach.

### Phylogenetic inference and demographic history

We reconstructed the phylogenetic relationship between populations using the nucleotide sequences extracted from the matrix of genome-based SNP genotypes. For this analysis, we applied a more relaxed genotype filtering option in VCFtools allowing up to 50% of missing genotypes per SNP (-max-missing-count 18), and kept only SNPs outside the repeat-rich regions identified during the annotation step. We then used MVFtools^56^ to convert the genotypes to an alignment of nucleotide sequences coding the heterozygotic positions with IUPAC ambiguity codes and also adding the reference sequence to the alignment. The resulting alignment of 675 767 bp of 34 (32 ingroup, 2 outgroups) terminals was used for coalescent phylogenetic inference with CASTER *v*. 1.19^57^ using the site patterns option with default settings but using ambiguity codes information and 16 rounds of placement and subsampling.

Strong population structure can arise through the combined effects of selection and genetic drift (i.e. fixation of random or not naturally selected alleles), particularly under conditions of geographic isolation, reduced gene flow, or demographic fluctuations^58^. To disentangle these processes, we reconstructed the demographic and migration history of the study populations. We used ANGSD* v*. 0.938^59^ to estimate per-site genotype likelihoods (*doSaf − 1*) for each ancestral population independently, using the GATK-based genome alignments as input. The SNPs were filtered using the following criteria: minimum – maximum depth: 1-50x, sites had to be covered in all individuals within each population; minimum mapping quality of 20; exclusion of incorrectly mapped, non-uniquely mapped reads; and retention of only correctly mapped paired-end reads (only_proper_pairs). To avoid potential confounding effects from loci under selection, we removed all protein-coding sites using the –sites and –rf options. We then used the realSFS tool of ANGSD to estimate the folded site frequency spectrum (SFS, i.e.: distribution of allele frequencies across all SNPs in one population). The resulting folded SFS estimates of each population were used as input for Stairway Plot *v*. 2.1^60^ to infer the demographic history, employing 200 bootstrap replicates of the SFS and assuming a mutation rate of 1e − 9 mutations per site per year^61–63^ and a generation time of two years^64^.

To identify populations that might have received migrants and thus genetic material from others, we used the LD-pruned SNP dataset and reconstructed drift trees using the software TREEMIX *v*. 1.13^65^. We ran 20 iterations of the algorithm allowing 0 to 5 migrations events (M) between tree edges and used the R-package *OptM*^66^ to infer the optimal M value. Samples from PAL were used to root the tree following the phylogenetic reconstruction results.

### Selection analyses

Natural selection and genetic drift might contribute to differences in allele frequencies among populations. Here we aimed to identify loci more likely associated with color-related selection rather than stochastic divergence. To do that, we conducted selection analyses using a predefined population division between red and green frogs. Three complementary selection scan methods were applied to identify positively selected loci: BayeScan^67^, Ohana^68^, and PCAdapt^69^.

BayeScan uses Bayesian inference combined with the Markov Chain Monte Carlo (MCMC) algorithm to estimate population allele frequencies and therefore detect outlier loci under selection pressures across populations^67^. We used the function *create bayescan input* from the *SambaR* package and PGDSpider *v.* 3.0.0.0^70^ to convert the PLINK files to the required BayeScan *geste* format. BayeScan was executed using default parameters (*n* = 5000, thin = 10, nbp = 20, pilot = 5000, burn in = 50 000, pr_odds = 10) to identify SNPs under positive, negative and neutral selection between color morphs of *O. granulifera* by comparing all individuals within and among populations of the same and different color phenotypes. On the other hand, Ohana is a maximum likelihood approach designed to identify genome regions under positive selection. It uses a Gaussian approximation to model changes in allele frequency, while accounting for admixture between populations and estimates a tree topology and uses it to find deviating genotypes^68^. In this case, we were interested in the tree branch that brings together the two green populations with the red population from San Josecito. We considered candidate loci showing signatures compatible with positive selection, those with the logarithm of likelihood ratios above the 99th percentile of the distribution (top 1% log-likelihood ratios). Lastly, PCAdapt can handle admixed individuals and does not require grouping individuals to populations, can handle missing and pooled sequencing data, and it is based on Principal Component Analysis (PCA) that measures the percentage of variation of a SNP explained by the first *K* principal components^69^. Single nucleotide polymorphisms with significant q-values (q ≤ 0.05) were considered candidate SNPs showing signatures compatible with positive selection in the BayeScan and PCAdapt analyses (considering only PC1, which differentiated green from red populations). The Tajimas´D statistic^71^ was calculated as part of the function *calcdiversity* from the *SambaR* package, to provide an overview of undergoing selection events in each population. Outlier SNPs annotation was performed using SNPdat *v*. 1.0.5^72^. Overlap among SNPs detected by the three selection methods was assessed using the *VennDiagram* package in R, and downstream analyses focused primarily on loci supported by all three methods. BayeScan, Ohana and PCAdapt are based on different statistical frameworks, differ in sensitivity and false-positive discovery rates, and vary in their power to detect selection signals across species and sampling designs. Therefore, none of them in isolation can fully capture the genomic signal of interest in most cases, and using their overlap provides a more conservative set of candidate SNPs with stronger support for selection. Results obtained using the reference genome were compared with those derived from the superTranscriptome.

### Coloration genes and over-representation analyses

To identify coloration-related genes, we cross-referenced our annotated results with previously published data on genes associated with the melanin synthesis pathway, carotenoid metabolism, pteridine synthesis, and iridophore guanine synthesis, as well as other coloration-related genes with diverse functions in multiple vertebrate species^73–76^. The molecular mechanisms underlying color production in amphibians, especially poison frogs, are not well understood. Therefore, the a priori list of color-related genes we used here is likely not comprehensive, yet it might provide first insights about color-related genes under selection in our study species. In addition, to characterize the functional significance of both sets of candidate genes under selection, we performed a Gene Ontology (GO) term over-representation analysis (ORA) of these genes using the *genekitr* package^77^. The human gene set, obtained from monthly-updated gene set libraries using the package *geneset*^78^ was used as background.

## Results

### Population genetics

The obtained superTranscriptome assembly includes 120 172 transcripts concatenated without redundancy into 18 886 supertranscripts. Phylogenetic analyses placed, with high confidence, the southernmost population PAL as basal for the species and sister to a clade formed by the red population SAN and a cluster including the ESQ and DAM populations (Fig. 1B). Genome-based (142 234 SNPs) and superTranscriptome-based (208 993 SNPs) PCAs were largely congruent in revealing two main genetic clusters (PC1): one composed of the two green populations, and another separated for the red populations (Fig. 1C, D). A further separation of the two red populations is evident in PC2. When using subsets of neutral SNPs (genome: 51 195; superTranscriptome: 70 187) similar clusters were observed, however, the proportion of explained variance in PC1 increased by more than 10% (Supplementary Fig. S1). Admixture analysis showed the best-fit *K* value for two ancestral populations when using the full dataset (Fig. 1E, F; Supplementary Fig. S2). However, when using only neutral SNPs, the best-fit *K* solution ranged between 2 and 3 ancestral populations (Supplementary Fig. S2).

Estimates of population genetic diversity are provided in Supplementary Table S2. The genome-based estimates indicate that the red population SAN has a slightly higher nucleotide diversity (*π* = 0.00309) than the rest (minimum – maximum *π*: 0.00265–0.00278). In terms of population-adjusted mutation rate (Watterson’s theta), SAN shows a slightly higher estimate (*Θ* = 0.00292) and PAL the lowest (*Θ* = 0.00243). Pairwise *Fst* between populations varied from 0.032 to 0.244, and Nei genetic distance ranged from 0.031 to 0.135. For both estimators the most pronounced divergence was found between the red population PAL and each of the two green populations (DAM and ESQ), while the red population SAN showed intermediate divergence from both PAL and green populations (Supplementary Table S3, Supplementary Table S4). These estimates were all extremely similar to those obtained when using the species superTranscriptome as reference.

**Fig. 2:**
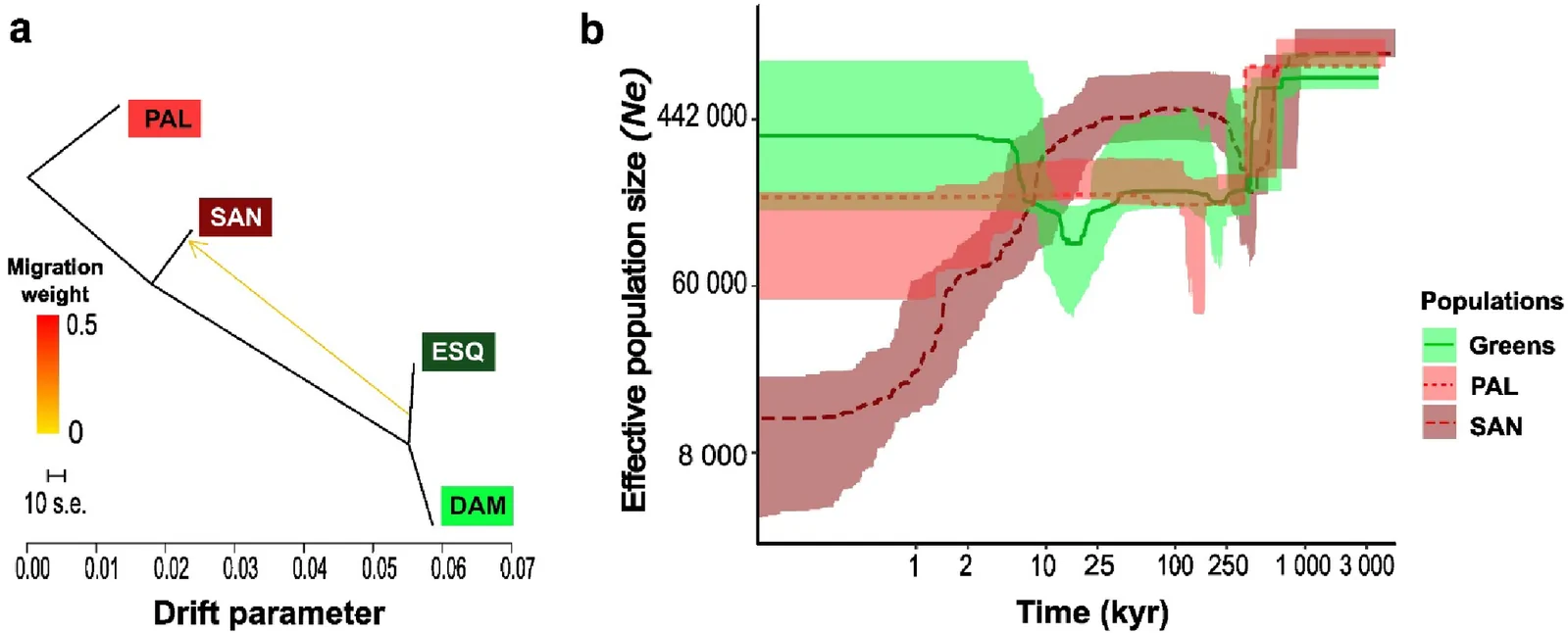
Estimations of migration and population size dynamics in *O. granulifera*. (**A**) Drift tree depicting the direction of the single migration event detected (arrow) by TREEMIX analyses. (**B**) Stairway plot depicting the change in mean (lines), and 75% confidence intervals (shades), of effective population size by population through time (in kyr: thousands of years ago). Both axes in (**B**) are log scaled.

A model with a single migration event stood out as the most likely in TREEMIX analyses with a migration edge connecting the ancestors of the ESQ population with current SAN individuals (Fig. 2A). Demographic inferences discarded the existence of severe bottlenecks in the history of the studied populations. Estimates of population sizes suggest that after a widespread sharp and brief decline of all populations 1000 - 500 thousand of years ago (kyr), the green population cluster (localities DAM and ESQ) and the red population of PAL have remained relatively stable. In SAN, however, a decline in population size is identifiable since around 25 kyr (coinciding with the Last Glacial Maximum) towards the present (Fig. 2B).

### Signatures of selection

The average Tajima´s D values for all populations and both approaches were positive, ranging from 0.007 to 0.029 for the genome mapping, and from 0.021 to 0.037 for the superTranscriptome mapping, with highly significant *P*-values (*P* < 0.01) in all calculations. This indicates that there is an overall lack of rare alleles in these populations, suggesting population contraction or balancing selection (Supplementary Table S2).

**Fig. 3:**
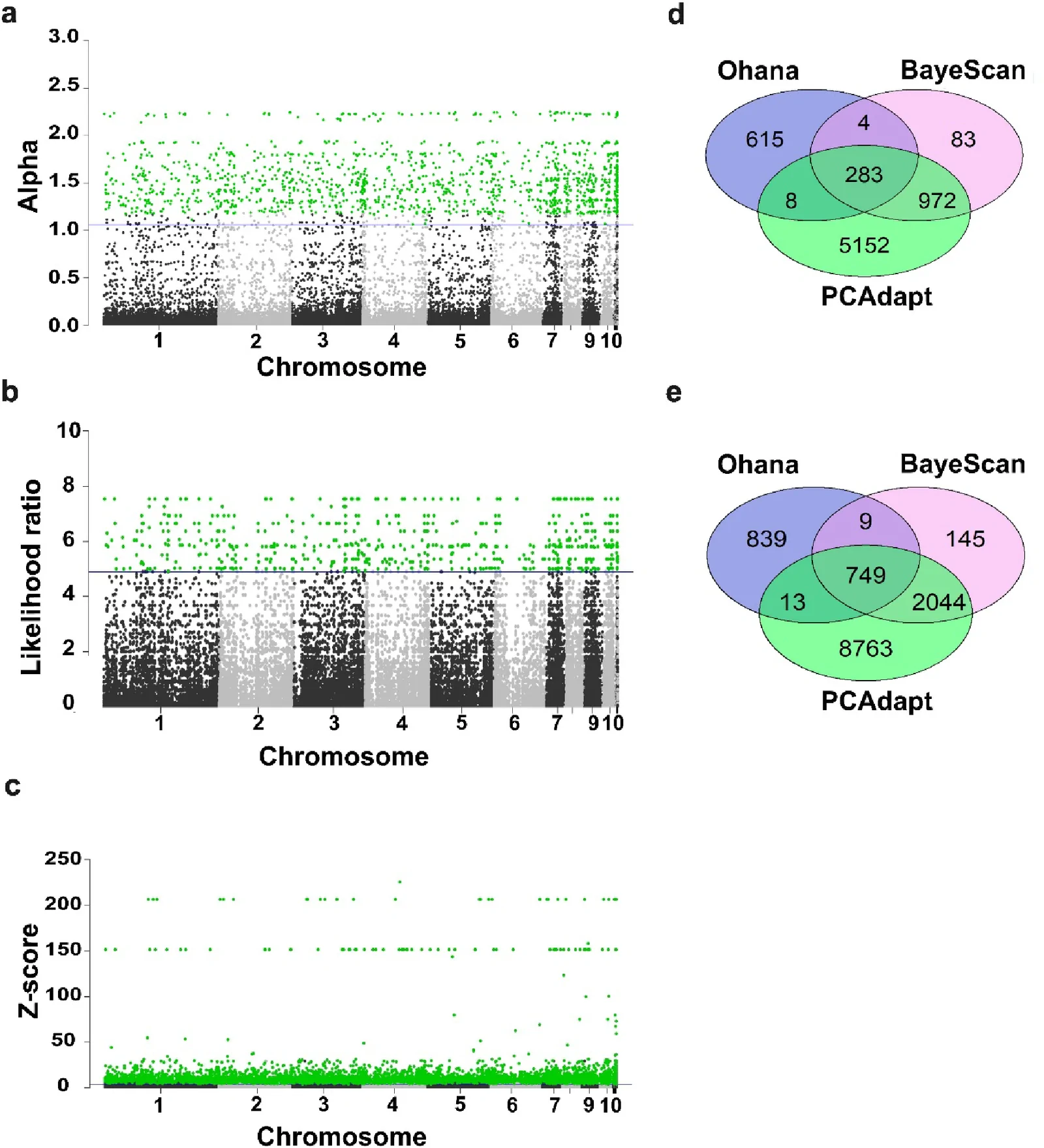
Candidate SNPs showing signatures compatible with positive selection. **A–C** Manhattan plots showing the distribution of outlier SNPs (green dots) identified by the three selection scan methods in the genome-based approach: (**A**) BayeScan, (**B**) Ohana, and (**C**) PCAdapt. **D**, **E** Venn diagrams showing the number of outlier SNPs detected by the three selection scan methods using a genome approach (**D**: 283 shared SNPs), and a superTranscriptome approach (**E**: 749 shared SNPs). From PCAdapt results, only the SNPs associated to the PC1 (red vs. green) where used for this analysis. In (**C**) the absolute values of z-scores are represented.

When using the genome-based approach, BayeScan identified 1342 candidate SNPs showing signatures compatible with positive selection between geographically structured populations with contrasting phenotypes, while Ohana identified 910 SNPs, and PCAdapt found 9362 SNPs, of which 6415 were associated with PC1 (i.e. differentiated between green and red frogs; Fig. 3A–C). Across the three methods, 283 SNPs were consistently detected (Fig. 3D). Of these 283 common SNPs, 281 could be mapped to annotated features of the *O. sylvatica* genome. Specifically, 104 SNPs were located within the “genes” features (corresponding to 104 genes, 97 with functional annotation, 7 without functional annotation), and from these 35 were synonymous substitutions and 69 were non-synonymous, while the other 177 SNPs were in intergenic regions (162 with functional annotation, 15 without functional annotation). Among the 104 genes containing candidate SNPs, nine corresponded to genes with known roles in coloration. All intergenic SNPs were located within 1 Mb of the nearest gene, and 11 were in proximity to genes with coloration-related functions.

When using the superTranscriptome-based approach, BayeScan identified 2947 candidate SNPs showing signatures compatible with positive selection, while Ohana identified 1610 SNPs, and PCAdapt identified 16 563 SNPs, of which 11 569 are associated with PC1. The three methods identified consistently 749 SNPs (Fig. 3E). These 749 outlier SNPs occurred in 735 functionally annotated genes, 219 SNPs were synonymous substitutions, while 530 SNPs were non-synonymous. Among the 735 genes, 49 had previous link to coloration in other taxa. Details on candidate SNPs showing signatures compatible with positive selection, their annotation, associated genes, and potential links to coloration are provided in Supplementary Table S5.

### Coloration genes and over-representation analyses

Among the genes containing candidate SNPs identified by both approaches (genome and superTranscriptome), 16 had previously been linked to coloration-related processes (*ADAR*, *BCO1*,* CBL*, *COPA*, *DVL2*, *FZD7*, *GMPPB*, *GNAI1*,* HTRA1*, *PICALM*, *PLXNB2*, *POGLUT1*, *SLC16A2*, *TET3*, *TRAF6*, *TSC2*). In addition, four coloration-related candidate genes (*GPATCH3*,* IMPDH1*,* INPP5B*,* MDM2*) where uniquely identified in the genome-based approach, whereas 33 where exclusively detected using the superTranscriptome-based approach (*PSAT1*,* MYCBP2*,* RETSAT*,* FLNA*,* SHROOM2*,* MPND*,* WNT3A*,* SYTL2*,* MDN1*,* ATRN*,* CBS*,* STX3*,* DNM2*,* COP1*,* NUP88*,* MEN1*,* CSF1R*,* MFSD12*,* CLCN7*,* PPP4C*,* ATE1*,* DSG4*,* RARG*,* PKN2*,* TFAP2A*,* TPCN2*,* IDH2*,* MAGOH*,* ALDOA*,* SPIRE1*,* WIPI1*,* CTR9*,* DYNLT3*) (Supplementary Table S5).

In the genome-based approach, 34.51% of the genes containing candidate SNPs showing signatures compatible with positive selection were annotated with at least one GO term. Among these, over-representation analysis revealed 19 enriched GO categories, including two molecular functions, 13 cellular components, and four biological processes (Supplementary Table S6). In the superTranscriptome-based approach, 36.73% of the genes containing candidate SNPs showing signatures compatible with positive selection were annotated with at least one GO term, yielding 30 enriched GO categories comprising 10 molecular functions, 15 cellular components, and five biological processes (Supplementary Table S7).

Over-represented molecular functions in the genome-based approach included histone modifying activity and cadherin binding. In contrast, the superTranscriptome-based approach revealed a broader range of enriched molecular functions, such as DNA binding, vitamin binding, phospholipid binding, GTPase binding, cadherin binding, lipoprotein particle receptor activity, and oxoglutarate-dependent dioxygenase activity. Enriched GO categories for both approaches were associated with cellular components including podosomes, desmosomes, endosomes, and the membranes and lumen of granule, vesicles and lysosomes. Regarding biological processes, the genome-based approach showed enrichment in regulation of cell-cell adhesion, cellular response to hydrogen peroxide, protein localization to the plasma membrane, and protein localization to the cell periphery. Meanwhile, the superTranscriptome approach highlighted processes such as regulation of neuron projection, endocytosis, response to ketone, establishment of organelle localization, and Golgi vesicle transport. A graphical summary of these over-represented categories is provided in Supplementary Fig. S3.

## Discussion

Single Nucleotide Polymorphisms have become the most widely used markers in population genetics, particularly for non-model organisms^79^. The high density of markers in each individual allows for detailed characterization of population genetic diversity and structure, even with a relatively small sample size^80^. In this study, we employed SNPs to examine the genetic structure and diversity of *O. granulifera*, and to identify candidate loci showing signatures compatible with positive selection between geographically structured populations with contrasting cryptic and aposematic phenotypes.

Gaining insight into the population structure and genetic diversity of a threatened species is essential for recognizing distinct conservation units and formulating targeted conservation strategies. Principal Component Analysis revealed a similar structure for the four analyzed datasets (genome vs. superTranscriptome, all SNPs vs. only neutral SNPs), where a clear differentiation between red and green populations was observed, as well as divergence between red populations. Our results regarding population structure and phylogeography are consistent with those of Brusa et al.^21^, who recognized two clades within the species, a southern clade of only red populations, and a northern clade that comprises green, yellow and red populations. Our sampled populations (DAM, ESQ, SAN, PAL) fall within the northern clade described by these authors, and similarly to their findings, PAL diverges from the remaining populations. According to that study, PAL belongs to the mitochondrial cyt b/16S northern clade, but nuclear/microsatellite markers showed an intermediate genetic structure where PAL shares genotypes from both north and south clusters. Based on that data, we hypothesize that the high divergence of PAL from the other populations might in part be the result of either mitochondrial introgression from the north or nuclear introgression across the river Térraba from the southern clade^21^. However, this hypothesis was not directly tested in the present study, as southern populations were not included in our analyses. In agreement with previous studies, our phylogenetic tree also suggests that the green morph derived directly from a red ancestor^21,23^.

The pronounced population structure observed indicates that genetic drift has likely contributed to genome-wide divergence among populations. Demographic events like bottlenecks (i.e. sharp reduction in population size due to extreme events such as environmental disasters, disease outbreaks, human activities), and migration (i.e. gene flow) typically reduce genetic differentiation among populations^81^. In geographically structured systems with limited gene flow, fixed or highly differentiated alleles may arise through stochastic processes rather than adaptive evolution alone. However, our demographic analyses found no evidence of severe bottlenecks, and the single historical migration event occurred between red and green populations, which lends support to the role of natural selection, likely determined by differing predation pressures^22^, in shaping the observed changes in allelic frequencies between color phenotypes. A similar reasoning led Bertram^82^ to identify positive selection, rather than neutral forces or migration, as the primary source of allelic frequency changes between wild and laboratory populations of the fruit fly *Drosophila* spp. Another study found that despite the existence of gene flow, natural selection shapes the evolution of the white clover *Trifolium repens* in response to urbanization^83^. Contrarily, historical demographic processes together with selection shape the complex patterns of differentiation along a latitudinal gradient among populations of the moor frog *Rana arvalis*^84^. Additionally, in humans, gene flow and drift, rather than selection, appear to be the primary determinants of recent genome-wide allele frequency change^85^. Together, these examples underscore that phenotypic diversity typically results from the interplay between neutral and selective mechanisms rather than selection alone, although the latter might play the key role in diversification for many cases.

Despite considerable progress has been made in identifying candidate genes linked to coloration in vertebrates^73,76^, including poison frogs^86,87^, relatively few studies have examined whether these genes harbor signatures of positive selection^10,16,88,89^. In this study, we identified candidate SNPs showing signatures compatible with positive selection that mapped to genes previously implicated in pathways related to carotenoid metabolism, melanin synthesis, pteridine synthesis, iridophore development, and coloration variation, and therefore represent candidate loci potentially involved in color divergence in *O. granulifera*. Our findings are consistent with a polygenic regulation of color polymorphism in poison frogs, where outlier SNPs, found in color and non-color related genes, play a crucial role. However, the interpretation of these candidate loci should be taken with caution because color phenotypes are not independent of geography and population history in our sampling design, and therefore some outlier SNPs identified in red versus green comparisons may reflect historical population structure, genetic drift, migration, introgression, or local adaptation unrelated to coloration rather than color divergence itself. Below we discuss genes that contain SNPs showing signatures compatible with positive selection, and that have been previously associated with coloration in other taxa, but future functional and comparative studies are needed to confirm their roles as determinants of color variation in *O. granulifera*.

Melanin, one of the most ancient and widespread pigments in nature, contributes to dark hues ranging from black to brown, as well as to darker shades of multiple colors generated through interactions with other pigments^90^. We identified candidate SNPs showing signatures compatible with positive selection between red and green populations in three genes previously associated with melanogenesis (*DVL2*, *FZD7*, and *GNAI1*). These genes participate in melanin synthesis regulation through the Wnt/β-catenin and PKA signaling pathways leading to β-catenin accumulation and activation of MITF, a key transcription factor for tyrosinase expression^91^. All three genes have previously been associated with pigmentation differences in vertebrates, including fishes^92,93^, birds^94,95^, mammals^96^, and poison frogs^16^. In particular, *GNAI1* was upregulated in red and green compared to blue frogs in *O. pumilio*^16^, making these loci promising candidates for future studies of coloration in our target species.

Pteridines are purine-based pigments stored in pterinosomes within xanthophores^9^, and are responsible for yellow, orange, and red hues in many taxa^97^. We identified candidate SNPs showing signatures compatible with positive selection in *MYCBP2*, a gene involved in pteridine biosynthesis^98,99^ that has been linked to pigmentation across species, including pale and yellow ventral coloration in the common lizard *Zootoca vivipara*^100^. However, in the same populations (and individuals) studied here, xanthopterin (i.e. a pteridine pigment) concentration was higher in skin than liver, but no significant morph differences were detected after accounting for tissue effects^30^.

Iridophores are specialized pigment cells responsible for producing structural coloration through stacks of guanine crystals that reflect different wavelengths of light^101^. In poison frogs, platelets thickness, along with pigmentation, contributes to skin coloration, with thinner crystals reflecting shorter wavelengths and producing blue hues, whereas thicker crystals reflect longer wavelengths, resulting in orange or red colors^102^. We identified candidate SNPs showing signatures compatible with positive selection in two genes (*IMPDH1* and *PSAT1*) involved in purine and guanine biosynthesis, processes relevant to the formation of guanine platelets in iridophores^103–107^. Both genes have previously been associated with coloration in poison frogs. In the mimic poison frog *Ranitomeya imitator*, *IMPDH1* was upregulated in orange and red skin patches in comparison to black patches^108^, whereas *PSAT1* was highly expressed in the skin of blue *O. pumilio* compared to red or green morphs^16^. In our studied populations, however, *PSAT1* was upregulated in red compared to green frogs^30^. In addition, in *O. granulifera* both red and green frogs lack organized iridophore layers, and when present, iridophores are sparse and commonly wrapped around by melanophores in red frogs, or the layer is discontinuous in green frogs^109^. Therefore, although *IMPDH1* and *PSAT1* are involved in guanine metabolism, they may contribute to other physiological processes or be associated with coloration in skin regions where iridophores are present.

Carotenoids are dietary pigments that contribute to the production and maintenance of vibrant yellow, orange, and red coloration^102,110,111^. As in other anurans^112–114^, *O. granulifera* gets its red coloration from consumed carotenoids, including keto-carotenoids and β-carotenes^30^, making genes involved in carotenoid metabolism particularly relevant candidates for color variation in this species. We identified candidate SNPs showing signatures compatible with positive selection in three genes (*BCO1*,* RARG*, and *RETSAT*) involved in carotenoid metabolism.The *BCO1* gene cleaves carotenoids into smaller compounds such as retinal^115^ and shows variable expression levels in multiple tissues across vertebrates, including fishes^116,117^, reptiles^118^, and amphibians. For instance, *BCO1* was highly expressed in green individuals of *O. pumilio* compared to red and blue morphs^89^, and in our studied species was upregulated in green in comparison to red populations^30^. On the other hand, *RARG* encodes a nuclear receptor involved in retinoic acid synthesis^119^, which plays a crucial role in regulating gene expression associated to cell growth, differentiation^120^, and pigmentation^121^. Additionally, *RETSAT* encodes an enzyme that participates in retinol metabolism^122^ by catalyzing the conversion of retinol to 13, 14-dihydroretinol^123^, and has been associated with pigmentation variation in other poison frogs, including yellow dorsal coloration intensity in *R. imitator*^88^.

In our study, most genes containing candidate SNPs showing signatures compatible with positive selection were not associated with any GO term, likely reflecting the limited functional annotation available for non-model organisms. Furthermore, GO enrichment analyses relied on human orthologs as a reference background and should be interpreted with caution, as cross-species annotation bias and incomplete annotations may affect the recovered categories. Consequently, the GO results presented here should be viewed as exploratory rather than definitive. Among the genes associated with GO terms, the majority have no direct link to coloration. In general, these genes are involved in multiple key functions such as cell signaling, growth and differentiation, vesicle composition and transport, cytoskeletal dynamics, metabolism, DNA binding, and ion transport^124^. However, those genes particularly involved in vesicle composition and transport could be indirectly relevant to pigmentation because pigments are stored and trafficked within intracellular vesicles. Overall, these results suggest that coloration differences in *O. granulifera* may involve multiple interacting cellular processes, although additional functional studies will be necessary to establish the specific roles of these candidate loci in phenotypic diversification and adaptation.

From a methodological perspective, the similar estimates of population structure and genetic diversity obtained using genome-based and superTranscriptome-based mapping of RNA-seq reads can be attributed to several factors. First, many genes involved in core cellular functions and adaptive processes are highly conserved across species^125^, allowing RNA-seq reads from *O. granulifera* to align reliably to the *O. sylvatica* genome. In addition, the superTranscriptome captures most expressed genes, and provides a high-quality species-specific reference, thereby minimizing alignment bias. Consistent with this expectation, mapping RNA-seq data to the *O. granulifera* superTranscriptome increased mapping quality by approximately 10% and reduced missing data (Supplementary Table S8, Supplementary Table S9). Although RNA-seq-derived SNPs are inherently restricted to expressed regions and may be influenced by tissue-specific and allele-specific expression, transcript isoforms, mapping bias, and differences in tissue sampling among populations^126–128^, these factors appeared to have little effect on the broad patterns of population structure and genetic diversity recovered here. On the other hand, selection scans were more effective in identifying candidate SNPs showing signatures compatible with positive selection when using a superTranscriptome rather than a heterospecific genomic reference. Because transcriptomes represent expressed genes^129^, which are the primary targets of selection^130^, a species-specific superTranscriptome may improve the detection of functionally relevant species-specific adaptive variants^131^ that could potentially be missed out when using another species genome as reference. Our results are truly encouraging for future research and suggest that a de novo assembled superTranscriptome is a viable alternative for population genomic and selection studies in non-model organisms lacking high-quality reference genomes.

## Conclusion

By using high-throughput sequencing technologies, and genomic and transcriptomic tools, this study advances the understanding of the molecular mechanisms underlying color polytypism in dendrobatid frogs. Our analyses revealed that the two populations with green phenotype are genetically very similar and originated from a red ancestor, while the two red populations are genetically very distinct and only one of them (SAN) has exchanged migrants with the green cluster. We identified candidate loci showing signatures compatible with positive selection between geographically structured populations of contrasting color phenotypes. A small subset of these loci is involved in pigment synthesis (melanin, pteridines), carotenoids metabolism, iridophores development (guanine platelets), and color variation in other vertebrates, which makes them good candidates to explain color variation in our study species, although their specific role in coloration needs further validation. Our findings illustrate the complex genetic architecture of skin color phenotypes of poison frogs and demonstrate the value of transcriptomics in non-model organism’s research. Further studies exploring additional populations, color morphs, and environmental factors will enhance our understanding of how genetic and ecological forces interact to shape the evolution of coloration and other key features in poison frogs.

## Supplementary Information

Below is the link to the electronic supplementary material.


Supplementary Material 1



Supplementary Material 2



Supplementary Material 3



Supplementary Material 4


## Data Availability

The raw RNA sequences of *Oophaga granulifera* used in this study are archived in the NCBI (SRA Bioproject PRJNA1234489, accession numbers: SRR32643849 - SRR32643922). The reference genome assembly is available at NCBI (accession number: GCA_033576555.1), and its annotation is available on Zenodo (https://doi.org/10.5281/zenodo.17804089). The assembled superTranscriptome of *O. granulifera* and the four genotype files (full dataset vs. only neutral SNPs per reference) are available on Zenodo (https://doi.org/10.5281/zenodo.17805830). Scripts used for the bioinformatics processing of RNA reads and data analysis are available on GitHub (https://github.com/cajigas/granulifera_selection) and (https://github.com/cajigas/granulifera_SNPs).
