# Supplementary material for: Genomic diversification between aposematic and cryptic phenotypes of Oophaga granulifera (Anura: Dendrobatidae)
Source: Sci Rep. 2026 Aug 25;16:26949. doi: 10.1038/s41598-026-66654-3 (PMC13522351; doi:10.1038/s41598-026-66654-3)
Supplement: Supplementary file 1 [file 41598_2026_66654_MOESM4_ESM.pdf]

SUPPLEMENTARY MATERIAL

in support of the article:

Genomic diversification between aposematic and cryptic phenotypes of *Oophaga granulifera* (Anura: Dendrobatidae)

Anaisa Cajigas Gandia[1*], Heike Pröhl[1], Julia Metzger[2], Ariel Rodríguez[1*]

[1]Institute of Zoology, University of Veterinary Medicine of Hannover, Bünteweg 17, 30559 Hannover, Germany.

[2]Institute of Animal Genomics, University of Veterinary Medicine of Hannover, Bünteweg 17p, 30559 Hannover, Germany.

Important note: In this supplementary file, figures and tables are presented in the order in which they are cited in the main manuscript to ensure clarity and ease of reference.

Table S1. Mean ± standard deviation in million-reads (Mr) of uniquely mapped reads per tissue type within populations of *Oophaga granulifera.* N represents the sample size per tissue in each population.

**Population**	**Morph**	**Tissue**	**N**	**Genome (Mr)**	**SuperTranscriptome (Mr)**
DAM	Green	liver	8	13.58 ± 4.53	16.5 ± 5.7
		skin	8	16.88 ± 3.39	19.23 ± 3.98
ESQ	Green	eyes	5	11.27 ± 2.19	14.19 ± 2.69
		liver	8	12.32 ± 4.55	15.08 ± 5.45
		skin	8	13.95 ± 4.45	15.94 ± 5.04
PAL	Red	liver	8	18.47 ± 7.81	22.96 ± 9.67
		skin	8	20.55 ± 7.09	23.42 ± 8.05
SAN	Red	eyes	5	13.25 ± 3.55	16.81 ± 4.53
		liver	8	16.89 ± 7.95	20.39 ± 9.62
		skin	8	16.03 ± 4.07	18.01 ± 4.55

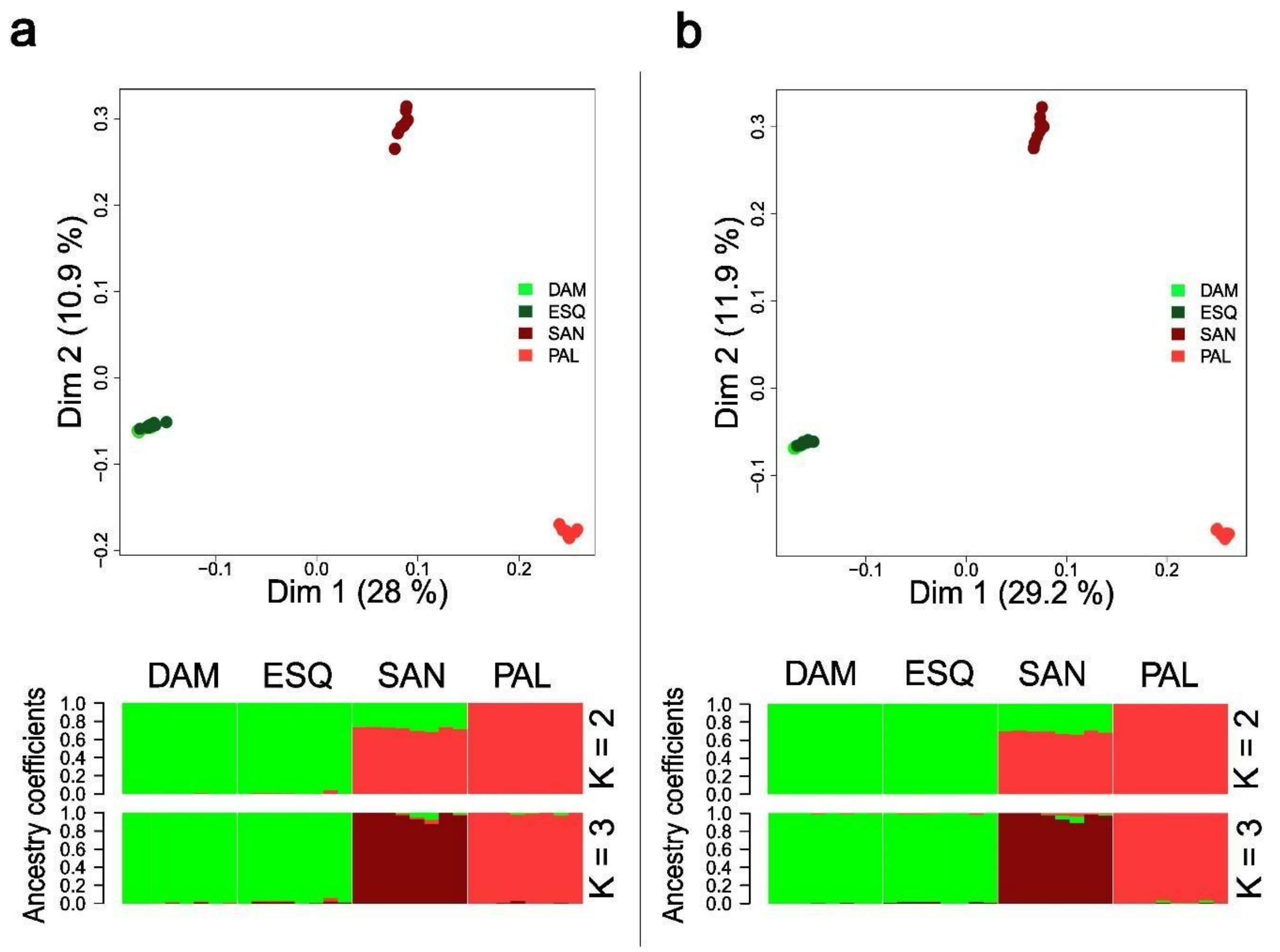

Figure S1. Population genetic structure of *O. granulifera* using only neutral SNPs identified by BayeScan. A) Principal Component Analysis and Admixture analysis: genome (N=51,195 SNPs). B) Principal Component Analysis and Admixture analysis: superTranscriptome (N=70,187 SNPs).

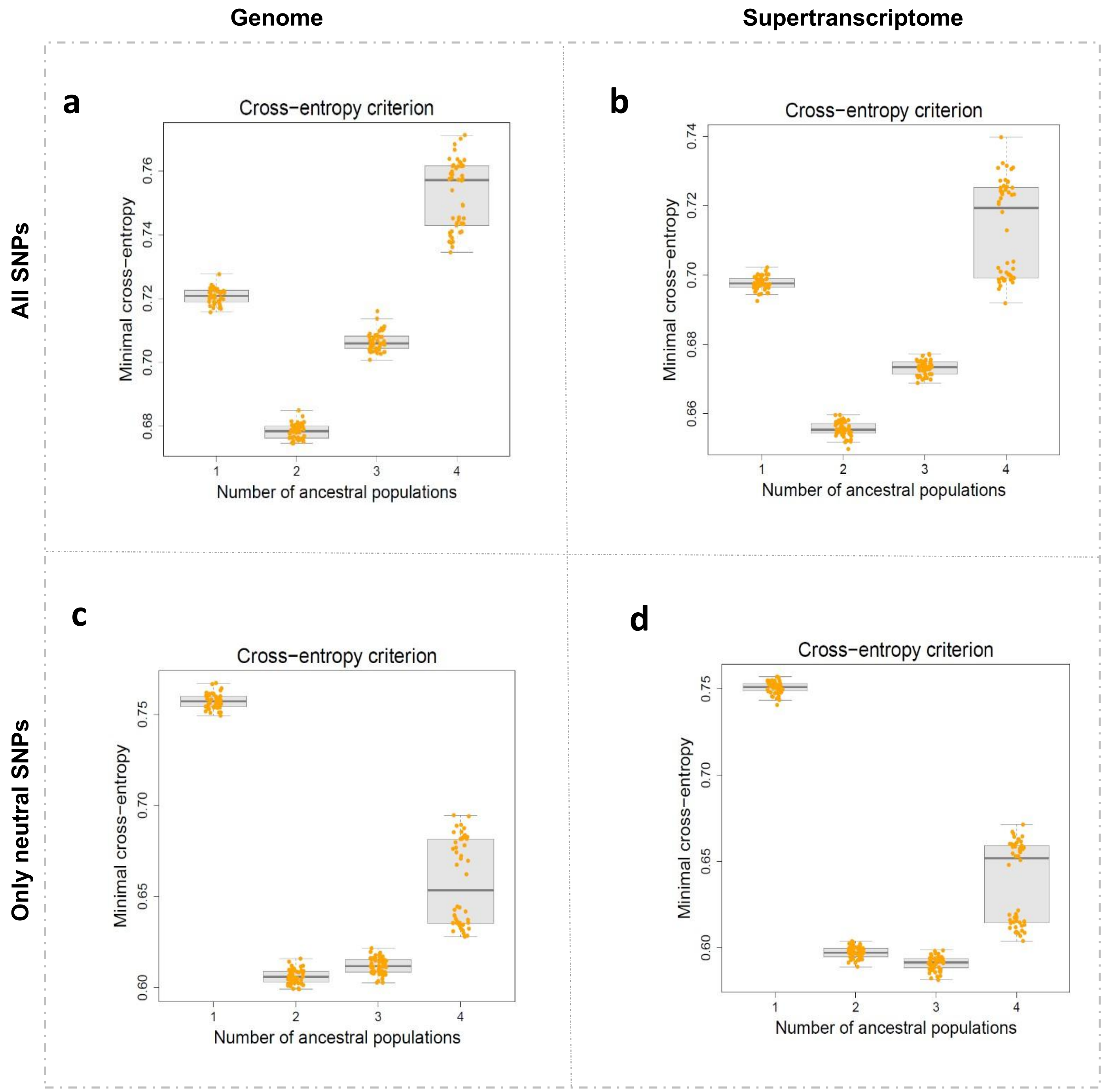

Figure S2. Cross-entropy criterion according to the *LEAce* function from *SambaR* package. Results show that the most likely *K* solution is between two and three.

Table S2. Population genetic diversity statistics given by SambaR. Genome/superTranscriptome. Minor allele frequency (MAF) scaled Multi-locus heterozygosity (MHLs), Nucleotide diversity (π), Watterson estimator theta scaled (Θs), Tajimas´D scaled (*D*s), (*): indicates same result for genome and superTranscriptome.

**Population**	**DAM**	**ESQ**	**SAN**	**PAL**
**MAF**	0.178 / 0.185	0.188 / 0.194	**0.210 / 0.214**	0.186 / 0.192
**MLHs**	0.936 / 0.944	0.986 / 0.995	**1.083 / 1.073**	0.983 / 0.981
**π**	0.00265 / 0.00275	0.00278 / 0.00288	**0.00309 / 0.00317**	0.00272 / 0.00281
**Θs**	0.00251 / 0.00253	0.00270 / 0.00263	**0.00292 / 0.00280**	0.00243 / 0.00244
***D*s**	0.007 / 0.021	0.008 / 0.025	0.019 / **0.037**	0.029 / **0.037**
p-value **X^2**	$p < 0.000001$* 25.11/327.07	$p < 0.000001$* 30.13/448.23	$p < 0.000001$* 443.85/1081.86	$p < 0.000001$* 147.28/938.93
**demography**	Contraction*	Contraction*	Contraction*	Contraction*
**selection**	Balancing*	Balancing*	Balancing*	Balancing*

Table S3. Pairwise Weir & Cockerham 1984 fixation index. Values in green cells correspond to the analysis made when using genome as reference, and in blue cells correspond to the analysis made using the superTranscriptome as reference.

	**DAM**	**ESQ**	**SAN**	**PAL**
**DAM**	0.000	0.032	0.146	**0.244**
**ESQ**	0.032	0.000	0.133	**0.234**
**SAN**	0.140	0.127	0.000	0.133
**PAL**	**0.238**	**0.230**	0.132	0.000

Table S4. Pairwise Nei genetic distance between populations. Values in green cells correspond to the analysis made when using genome as reference, and in blue cells correspond to the analysis made using the superTranscriptome as reference.

	**DAM**	**ESQ**	**SAN**	**PAL**
**DAM**	0.000	0.031	0.088	**0.135**
**ESQ**	0.030	0.000	0.084	**0.134**
**SAN**	0.083	0.079	0.000	0.082
**PAL**	**0.133**	**0.132**	0.080	0.000

Table S5. Single nucleotide polymorphisms showing signatures compatible with positive selection among red and green populations of *O. granulifera* when using a reference genome and superTranscriptome. For each SNP entry, we show the reference used for mapping RNA reads, the region where the SNP is, chromosome and position, start and end of the current feature. For those occurring in intergenic regions the distance to the closest gene is shown, gene ID according to the reference, gene name and gene name description (only for coloration-related genes), as well as BayeScan metrics (*q-value*, *alpha*, F_{st}) along with the potential link to pigmentation of the gene and the literature source. Additionally, the strand sense, estimated reading frame, codon and amino acid, and whether the substitutions are synonymous or non-synonymous are shown. Please note that in this excel file, three different sheets appear: one called “All SNPs” where all candidate SNPs likely under positive selection identified in genome-based and superTranscriptome-based analysis appear. The second sheet is called “Only color

related genes” and here you can find the same information than in the first sheet but filtered to show just color related genes. The third sheet called “Shared genes G-STranscriptome” shows the color related genes that were identified when using both references. (Excel table S5).

Table S6. Gene ontology (GO) terms enriched in the over-representation analysis using package *genekitr* for the candidate genes showing signatures of selection in the genome approach. For each GO term, we show its description, gene ratio, background ratio, rich factor, fold enrichment, z-score, p-value, p-value adjusted, q-value, gene ID and symbol, as well as the number of genes and the ontology term. (CSV table S6).

Table S7. Gene ontology (GO) terms enriched in the over-representation analysis using package *genekitr* for the candidate genes showing signatures of selection in the superTranscriptome approach. For each GO term, we show its description, gene ratio, background ratio, rich factor, fold enrichment, z-score, p-value, p-value adjusted, q-value, gene ID and symbol, as well as the number of genes and the ontology term. (CSV table S7).

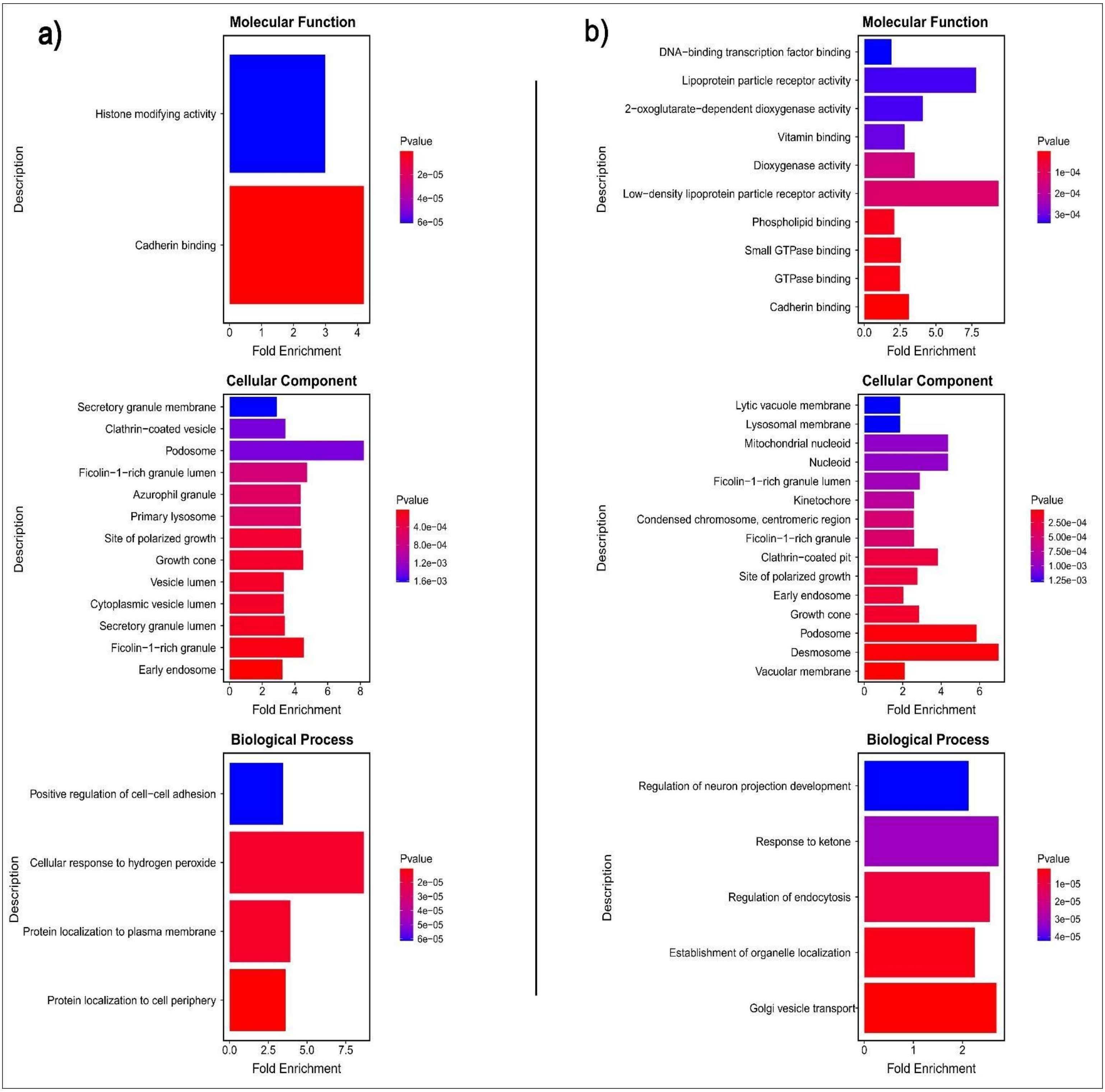

Figure S3. Gene Ontology (GO) term over-representation analysis for the genes with candidate SNPs showing signatures compatible with positive selection. A: genome (N=104 genes). B: superTranscriptome (N= 735 genes). GO terms as described by molecular function, cellular component and biological process are shown for each gene set.

Table S8. Comparison of mapping quality parameters between the genome and the superTranscriptome.

Parameter	Genome	SuperTranscriptome
mean mapped reads number	15565917.12	18475786.03
mean % uniquely mapped reads	54.51%	64.85%
minimum % uniquely mapped reads per sample	37.75%	44.49%
maximum % uniquely mapped reads per sample	67.45%	77.84%

Table S9. Summary statistics of SNPs retained for analyses after filtering and thinning, for both the genome and the superTranscriptome. In our case, the applied filters in *SambaR* were set to retain all SNPs (see Methods).

Parameter	Genome	SuperTranscriptome
Number of individuals	32	32
Number of retained SNPs	142 234	208 993
Mean spacing between SNPs	31812.89	202.8
Mean proportion of missing data per individual	0.08	0.06
GC content	0.50	0.51
Transition vs transversion ratio	2.26	2.44
